# Epacadostat plus pembrolizumab versus placebo plus pembrolizumab for advanced urothelial carcinoma: results from the randomized phase III ECHO-303/KEYNOTE-698 study

**DOI:** 10.1186/s12885-023-11213-6

**Published:** 2024-07-25

**Authors:** Irfan Cicin, Elizabeth R. Plimack, Howard Gurney, Raya Leibowitz, Boris Y. Alekseev, Francis X. Parnis, Avivit Peer, Andrea Necchi, Joaquim Bellmunt, Hiroyuki Nishiyama, Jason Clark, Mihaela Munteanu, Ritesh Kataria, Calvin Jia, Thomas Powles, Cora N. Sternberg

**Affiliations:** 1 Department of Medical Oncology, Trakya University, 22030 Edirne, Turkey; 2 Fox Chase Cancer Center, Philadelphia, PA USA; 3 Macquarie University, Sydney, NSW Australia; 4 Oncology Institute and Cancer Research Centre, Sheba Medical Centre Hospital, Tel Hashomer, Ramat Gan, Israel; 5 Sackler Faculty of Medicine, Tel Aviv University, Tel Aviv, Israel; 6 PA Hertsen Moscow Cancer Research Institute, Moscow, Russia; 7 Adelaide University and Adelaide Cancer Centre, Kurralta Park, SA Australia; 8 Rambam Health Care Center, Haifa, Israel; 9 Fondazione IRCCS, Istituto Nazionale Dei Tumori, Milan, Italy; 10 Beth Israel Deaconess Medical Center and PSMAR-IMIM Lab, Harvard Medical School, Boston, MA USA; 11 University of Tsukuba, Tsukuba, Japan; 12 Incyte Corporation, Wilmington, DE USA; 13 Merck & Co., Inc., Rahway, NJ USA; 14 Barts Health and the Royal Free NHS Trusts, Barts Cancer Institute, and Queen Mary University of London, London, UK; 15 Englander Institute for Precision Medicine, Weill Cornell Medicine, New York, NY USA

**Keywords:** IDO1, Epacadostat, PD-L1, Pembrolizumab, Urothelial carcinoma, Immune checkpoint inhibition, Randomized controlled study

## Abstract

**Background:**

Indoleamine 2,3-dioxygenase 1 (IDO1) levels correlate with poor outcomes in urothelial carcinoma (UC). IDO1 and programmed death-ligand 1 (PD-L1) are often co-expressed. Epacadostat is a potent and highly selective inhibitor of IDO1. In a subgroup analysis of patients with advanced UC participating in a phase I/II study, epacadostat-pembrolizumab treatment produced an objective response rate (ORR) of 35%.

**Methods:**

ECHO-303/KEYNOTE-698 was a double-blinded, randomized phase III study of adults with metastatic or unresectable locally advanced UC with recurrence or progression following first-line platinum-based chemotherapy. Participants were randomized to epacadostat 100 mg twice daily (BID) plus pembrolizumab or placebo plus pembrolizumab until completion of 35 pembrolizumab infusions, disease progression, or unacceptable toxicity. The primary endpoint was investigator-assessed ORR per Response Evaluation Criteria in Solid Tumors version 1.1.

**Results:**

Target enrollment was 648 patients; enrollment was halted early based on efficacy results from the phase III ECHO-301/KEYNOTE-252 study in metastatic melanoma. Forty-two patients were randomized to each treatment arm. Median duration of follow-up was 62 days in each arm. The investigator-assessed ORR (unconfirmed) was 26.2% (95% CI 16.35–48.11) for epacadostat plus pembrolizumab and 11.9% (95% CI 4.67–29.50) for placebo plus pembrolizumab. Two complete responses were reported, both in the placebo-plus-pembrolizumab arm. Circulating kynurenine levels increased from C1D1 to C2D1 in the placebo-plus-pembrolizumab arm and numerically decreased in the epacadostat-plus-pembrolizumab arm. The safety profile of epacadostat plus pembrolizumab was similar to that of pembrolizumab monotherapy, although a numerically greater proportion of patients in the combination vs. control arm experienced treatment-related grade ≥ 3 adverse events (16.7% vs. 7.3%). One patient in each arm died due to cardiovascular events, which were not deemed drug-related. No new safety concerns were identified for either agent.

**Conclusions:**

Epacadostat plus pembrolizumab demonstrated anti-tumor activity and was generally tolerable as second-line treatment of patients with unresectable locally advanced or recurrent/progressive metastatic UC. Epacadostat 100 mg BID, when administered with pembrolizumab, did not normalize circulating kynurenine in most patients. Further study of combined IDO1/PD-L1 inhibition in this patient population, particularly with epacadostat doses that result in durable normalization of circulating kynurenine, may be warranted.

**Trial registration:**

ClinicalTrials.gov, NCT03374488. Registered 12/15/2017.

**Supplementary Information:**

The online version contains supplementary material available at 10.1186/s12885-023-11213-6.

## Background

Bladder cancer is the tenth most common cancer globally, with an estimated 550,000 new cases and 200,000 deaths reported in 2018 [1]. Urothelial carcinoma (UC), which can arise from the bladder, renal pelvis, ureter, or urethra, accounts for the majority (> 90%) of cases of bladder cancer [2, 3]. Platinum-based chemotherapy is the preferred first-line treatment for locally advanced or metastatic UC [2, 3], but the disease progresses in most patients [4, 5]. The 5-year survival rate for metastatic disease is poor, with estimates ranging from 0 to 10% [6–8].

A number of immune checkpoint inhibitors targeting programmed cell death protein 1 (PD-1; eg, pembrolizumab, nivolumab) or programmed death-ligand 1 (PD-L1; eg, atezolizumab, avelumab, durvalumab) are approved for the treatment of patients with platinum-refractory advanced UC [2, 9]. Recently, avelumab has been approved in the post-chemotherapy switch maintenance space in patients not progressing to platinum chemotherapy [10]. In patients with advanced UC that has progressed following platinum-based chemotherapy, use of a single-agent checkpoint inhibitor is superior to single-agent chemotherapy, but the median overall survival (OS) remains less than 1 year and the response rate remains low (approximately 20%) [11–13]. In KEYNOTE-045, the only randomized study comparing PD-1 inhibition with chemotherapy in this setting, the superiority of PD-1 inhibition with pembrolizumab monotherapy persists over time [14, 15]. Thus, evaluation of second-line treatment options that can improve clinical outcomes in patients with platinum-refractory metastatic UC is of great interest. Novel therapeutic approaches including immunotherapy and targeted therapies (eg, enfortumab vedotin and sacituzumab govitecan) have recently demonstrated promising activity for relapsed disease and are likely to impact standard of care in the future [16, 17].

Cancer cells suppress anti-tumor immunity via multiple pathways [18], so combinations of unique and complementary targeted immunotherapies may help to further overcome tumor-mediated immunosuppression. Indoleamine 2,3-dioxygenase 1 (IDO1) is an enzyme that catabolizes tryptophan into kynurenine, and increased IDO1 activity results in tryptophan depletion and kynurenine accumulation. These effects suppress effector T-cell function and promote regulatory T-cell proliferation [19]. Increased levels of IDO1 have been correlated with disease progression and decreased rates of disease-specific survival in patients with UC [20]. IDO1, the expression of which is induced by interferon gamma [21], has been shown to have immunosuppressive effects [22], including countering the anti-tumor effects of immune checkpoint inhibitors [23].

A number of malignancies show coexpression of IDO1 and PD-L1 [24–27]. In preclinical mouse models, IDO1 inhibition has been demonstrated to synergize with PD-L1 inhibition in delaying tumor growth and prolonging survival [23, 28]. These findings may be due to preservation of anti-cancer inflammatory responses via IDO1 inhibition.

Epacadostat is a potent and highly selective inhibitor of IDO1. In a phase I study of patients with advanced solid tumors, treatment with twice-daily (BID) doses of ≥ 100 mg reduced plasma kynurenine levels to those observed in healthy subjects [29]. In the phase I/II ECHO-202/KEYNOTE-037 study (NCT02178722), patients with stage IIIB–IV or recurrent solid tumors received combination treatment with epacadostat and pembrolizumab. In a preliminary analysis of the subgroup of patients with advanced UC, epacadostat plus pembrolizumab resulted in an objective response rate (ORR) of 35% (13/37) and was generally well tolerated [30]. In light of these findings, the ECHO-303/KEYNOTE-698 study, which compared epacadostat plus pembrolizumab with placebo plus pembrolizumab in patients with unresectable locally advanced or recurrent/progressive metastatic UC for whom first-line platinum-based chemotherapy failed, was undertaken.

## Methods

### Study design and participants

ECHO-303/KEYNOTE-698 (NCT03374488) was an international, active-controlled, double-blinded, randomized phase III study. This study was conducted at 82 centers in 16 countries. Eligible adults (aged ≥ 18 years) had confirmed UC of the urinary tract that had progressed or recurred following one prior platinum-based chemotherapy regimen administered for the treatment of inoperable locally advanced or metastatic disease, had one or more lesions that were measurable per Response Evaluation Criteria in Solid Tumors version 1.1 (RECIST v1.1) [31], had an Eastern Cooperative Oncology Group performance status of 0–1, and provided tumor tissue for the central analysis of PD-L1. Patients were excluded if they had prior therapy with inhibitors targeting PD-1, PD-L1, PD-L2, or IDO1; agents directed against any other stimulatory or co-inhibitory T-cell receptor; or any other antibody or drug targeting T-cell costimulatory pathways in the adjuvant or advanced/metastatic setting.

### Treatment and procedures

Participants were randomized (1:1) to receive epacadostat plus pembrolizumab or placebo plus pembrolizumab until the completion of 35 pembrolizumab infusions (taking approximately 2 years), disease progression, unacceptable toxicity, or another discontinuation criterion was met. Randomization was stratified by Bellmunt risk score (0 vs. 1 vs. ≥ 2) [32] and PD-L1 expression (combined positive score [CPS] ≥ 10 vs. < 10) per immunohistochemistry. Pembrolizumab 200 mg was infused intravenously every 3 weeks, and epacadostat (or matching placebo) 100 mg was dosed orally BID. Dose reductions were permitted to mitigate immune-related adverse events (AEs), but re-escalation of epacadostat (or placebo) was not permitted. Blood for analysis of serum kynurenine levels was drawn before dosing on day 1 of cycles 1 and 2 (C1D1 and C2D1, respectively) when patients were in a fasted state. Circulating kynurenine levels were determined at Worldwide Clinical Trials, Morrisville, NC, with a proprietary validated liquid chromatography tandem mass spectrometry assay using calibrated standards.

### Study conduct

The study was initiated on December 22, 2017. On May 2, 2018, a decision was made to permanently stop enrollment. This decision was based on the results of the phase III ECHO-301/KEYNOTE-252 study (NCT02752074), which compared epacadostat (100 mg BID) plus pembrolizumab with placebo plus pembrolizumab in patients with advanced melanoma [33]. The dual primary endpoints of ECHO-301/KEYNOTE-252 were progression-free survival (PFS) per independent central review and OS. During the second interim analysis, the external data monitoring committee concluded that PFS was not improved with combination therapy relative to pembrolizumab monotherapy and anticipated that OS would not reach statistical significance.

Following the halted enrollment, the ECHO-303/KEYNOTE-698 study was unblinded after the last patient enrolled had completed the week 9 imaging assessment for the analysis of efficacy. Patients demonstrating clinical benefit based on investigator assessment were allowed to continue receiving treatment (open-label combination therapy or pembrolizumab monotherapy).

ECHO-303/KEYNOTE-698 was conducted in compliance with the Declaration of Helsinki, the International Council on Harmonization Guidelines for Good Clinical Practice, and applicable national and local regulatory requirements. The study protocol was approved by the Independent Ethics Committee/Institutional Review Board at each participating site, and all patients provided written informed consent.

### Endpoints

The original dual primary endpoints of ECHO-303/KEYNOTE-698 were PFS per independent central review and OS. When enrollment was stopped, the protocol was amended, and the primary endpoint was changed to investigator-assessed ORR per RECIST v1.1 based on all available imaging assessments after the last patient completed the week 9 + 14 day imaging assessment (due to the protocol amendment, study-mandated efficacy procedures, including transmission of images for central review, were discontinued). ORR was defined as the proportion of patients with a complete or partial response. As a secondary objective, safety was assessed throughout the study, with AEs coded per Medical Dictionary for Regulatory Activities version 21.0 and graded per Common Terminology Criteria for Adverse Events version 4.03. The pharmacodynamic activity of epacadostat was assessed as one of the exploratory objectives by evaluating change from baseline in circulating kynurenine levels.

### Statistics

The original target enrollment was 648 patients, but when enrollment was stopped, the target was revised to 85 participants. The intent-to-treat population, composed of all randomized patients, was used for the efficacy analysis. ORR was determined for each treatment group, and the corresponding 95% confidence intervals (CIs) were calculated using the Clopper-Pearson exact method. Although protocol-specified efficacy imaging was stopped at week 9, some patients had completed scans beyond week 9 at the time enrollment was terminated. Thus, ORR was assessed using all available imaging data at the time of cutoff, as well as data collected only at week 9. The safety population was composed of patients who received one or more doses of study treatment, with patients analyzed according to the treatment received. Safety outcomes were summarized using descriptive statistics. Circulating kynurenine levels were assessed among patients who provided blood samples on C1D1 and C2D1, and comparisons were conducted using a paired t-test within each treatment arm. The cutoff date for these analyses was August 15, 2018.

## Results

### Participants

**Fig. 1:**
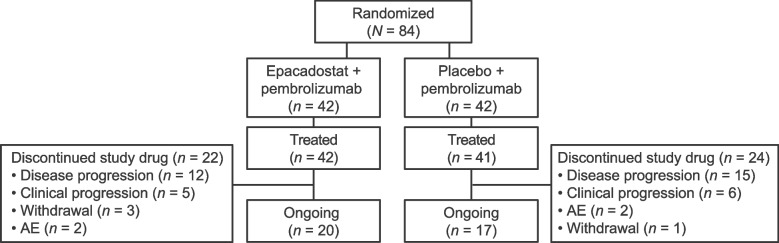
Patient disposition. AE, adverse event

A total of 84 patients with advanced UC were randomized (epacadostat plus pembrolizumab, *n* = 42; placebo plus pembrolizumab, *n* = 42) (Fig. 1). One patient randomized to placebo plus pembrolizumab was not treated because of withdrawn consent after randomization. In both treatment arms, the most common reason for study drug discontinuation was disease progression. Because patients with ongoing clinical benefit could have continued study treatment (per investigator discretion), 47.6% and 41.5% of treated patients were still receiving epacadostat plus pembrolizumab and pembrolizumab monotherapy, respectively, at data cutoff.

Patient characteristics were generally balanced between treatment arms, although the proportion of patients with prior neoadjuvant/adjuvant platinum-based chemotherapy was numerically higher in the epacadostat plus pembrolizumab arm (45.2%) than in the placebo plus pembrolizumab arm (26.2%) (Table 1). One-third of patients had a Bellmunt risk score ≥ 2, and approximately 60% had a PD-L1 CPS < 10. The majority of patients presented with visceral metastases (epacadostat plus pembrolizumab, 73.8% [31/42]; placebo plus pembrolizumab, 83.3% [35/42]). In the experimental arm, the median (range) duration of exposure to epacadostat and pembrolizumab was 98 (7–162) and 86.5 (1–150) days, respectively. In the control arm, the median (range) duration of exposure to placebo and pembrolizumab was 67 (10–168) and 65 (1–167) days, respectively. The median duration of follow-up was 62 days in each arm.

**Table 1:** Patient demographics and disease characteristics

	**Epacadostat + pembrolizumab** **(*n* = 42)**	**Placebo + pembrolizumab** **(*n* = 42)**
Male, *n* (%)	35 (83.3)	37 (88.1)
Age, median (range) (years)	69.5 (48–86)	67.5 (29–79)
Age ≥ 65 years, *n* (%)	27 (64.3)	24 (57.1)
Race, *n* (%)		
White	35 (83.3)	32 (76.2)
Asian	5 (11.9)	6 (14.3)
Unknown	2 (4.8)	4 (9.5)
ECOG performance status,^a^ *n* (%)		
0	20 (47.6)	18 (42.9)
1	22 (52.4)	24 (57.1)
Disease status at screening, *n* (%)		
Locally advanced/unresectable	7 (16.7)	2 (4.8)
Metastatic	35 (83.3)	40 (95.2)
Metastases location, *n* (%)		
Visceral disease	31 (73.8)	35 (83.3)
Lymph node only	11 (26.2)	7 (16.7)
Liver metastases present, *n* (%)	5 (11.9)	9 (21.4)
Primary tumor location, *n* (%)		
Upper tract	9 (21.4)	11 (26.2)
Lower tract	33 (78.6)	31 (73.8)
Prior neoadjuvant/adjuvant platinum-based chemotherapy, *n* (%)	19 (45.2)	11 (26.2)
Prior BCG therapy, *n* (%)	4 (9.5)	3 (7.1)
Bellmunt risk score, *n* (%)		
0	10 (23.8)	11 (26.2)
1	18 (42.9)	17 (40.5)
≥ 2	14 (33.3)	14 (33.3)
PD-L1 status, *n* (%)		
CPS ≥ 10	17 (40.5)	18 (42.9)
CPS < 10	25 (59.5)	24 (57.1)
Prior platinum-based chemotherapy, *n* (%)		
Cisplatin	22 (52.4)	24 (57.1)
Carboplatin	19 (45.2)	18 (42.9)
Other	1 (2.4)	0

*Abbreviations*: *BCG* Bacillus Calmette-Guérin, *CPS* Combined positive score, *ECOG* Eastern Cooperative Oncology Group, *PD-L1* Programmed death-ligand 1

^a^Assessed during screening

### Response rates

**Fig. 2:**
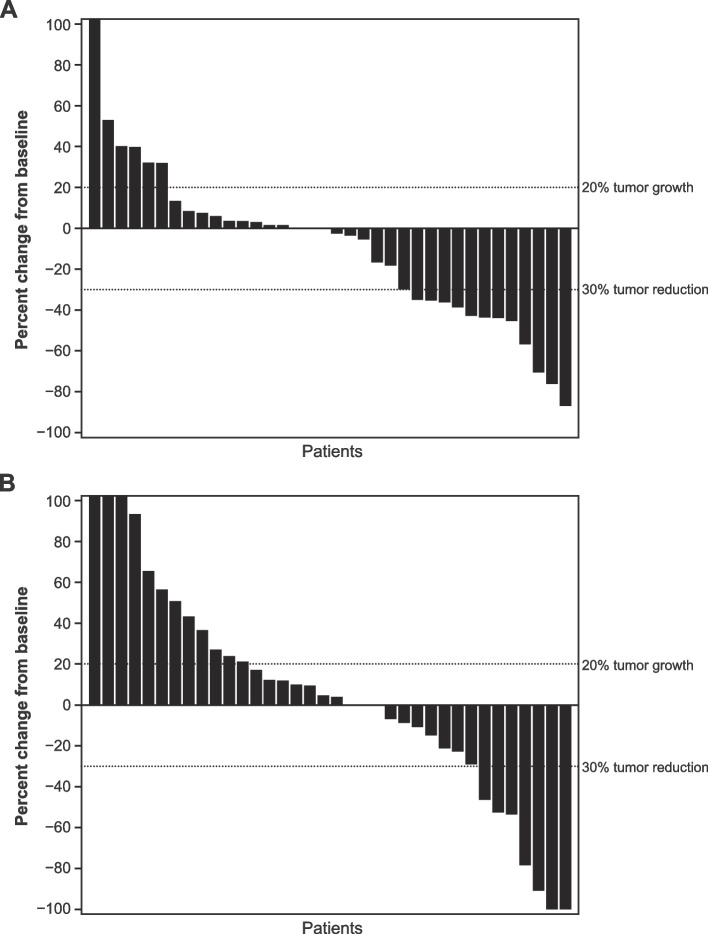
Best target lesion change from baseline based on investigator assessment per RECIST v1.1 (intent-to-treat population). **a** Epacadostat plus pembrolizumab. **b** Placebo plus pembrolizumab. RECIST v1.1, Response Evaluation Criteria in Solid Tumors version 1

Based on all available data at cutoff, ORR (unconfirmed, primary endpoint) was 26.2% (95% CI 16.35–48.11) for epacadostat plus pembrolizumab and 11.9% (95% CI 4.67–29.50) for placebo plus pembrolizumab (Table 2). No complete responses were observed in the epacadostat-plus-pembrolizumab arm; two were observed in the placebo-plus-pembrolizumab arm. Rates of progressive disease were 31.0% for epacadostat plus pembrolizumab and 52.4% for placebo plus pembrolizumab. The corresponding ORRs based on data from the week 9 visit only were 21.4% (95% CI 12.88–44.36) and 9.5% (95% CI 3.20–26.74) (Additional file 1: Supplementary Table 1). Waterfall plots summarize the best change in target lesion size from baseline using all available data at cutoff (Fig. 2) and data from the week 9 visit only (Additional file 2: Supplementary Fig. 1).

**Table 2:** Investigator-assessed best overall response per RECIST v1.1 (intent-to-treat population)^a^

*n* (%)	Epacadostat + pembrolizumab (*n* = 42)	Placebo + pembrolizumab (*n* = 42)
ORR^b^ [95% CI^c^]	11 (26.2) [16.35–48.11]	5 (11.9) [4.67–29.50]
Complete response	0	2 (4.8)
Partial response	11 (26.2)	3 (7.1)
Stable disease	12 (28.6)	9 (21.4)
Progressive disease	13 (31.0)	22 (52.4)
No assessment^d^	6 (14.3)	6 (14.3)

*Abbreviations*: *CI* Confidence interval, *ORR* Objective response rate, *RECIST v1.1* Response Evaluation Criteria in Solid Tumors version 1.1

^a^Based on all available data at cutoff; responses were unconfirmed

^b^Includes patients with an unconfirmed complete or partial response

^c^Per the Clopper-Pearson exact method

^d^Includes patients with a baseline but no post-baseline assessment, including those who discontinued or died before the first post-baseline scan

### Safety and tolerability

In total, 97.6% of patients treated with epacadostat plus pembrolizumab reported an AE, most commonly fatigue (28.6%), constipation (23.8%), and anemia (23.8%). A total of 90.2% of patients administered placebo plus pembrolizumab reported an AE, most commonly anemia (22.0%) (Table 3). Grade ≥ 3 AEs occurred at similar frequency in the two treatment arms (35.7% vs. 39.0%), but the frequency of treatment-related grade ≥ 3 AEs was numerically higher with epacadostat plus pembrolizumab (16.7%) than with placebo plus pembrolizumab (7.3%). The rate of serious AEs was similar in the two treatment arms (26.2% vs. 29.3%). Serious AEs reported in more than one patient in either treatment arm were urinary tract infection (epacadostat plus pembrolizumab, *n* = 2; placebo plus pembrolizumab, *n* = 3), ileus (epacadostat plus pembrolizumab, *n* = 2), and hematuria (epacadostat plus pembrolizumab, *n* = 2; placebo plus pembrolizumab, *n* = 1). Serious treatment-related AEs occurred only in the epacadostat-plus-pembrolizumab arm, with four events (amylase increased, ataxia, peripheral motor neuropathy, pneumonitis) reported in three patients. One patient in each treatment arm died (pulmonary embolism in the epacadostat-plus-pembrolizumab arm and myocardial infarction in the placebo-plus-pembrolizumab arm). Neither death was considered related to study treatment.

**Table 3:** Safety summary (as-treated analysis)^a^

Patients, *n* (%)	Epacadostat + pembrolizumab (*n* = 42)	Placebo + pembrolizumab (*n* = 41)
Any AE	41 (97.6)	37 (90.2)
Fatigue^b^	12 (28.6)	5 (12.2)
Constipation^b^	10 (23.8)	5 (12.2)
Anemia^b^	10 (23.8)	9 (22.0)
Decreased appetite^b^	9 (21.4)	4 (9.8)
Hematuria^b^	9 (21.4)	2 (4.9)
Treatment-related AE	24 (57.1)	22 (53.7)
Grade ≥ 3 AE	15 (35.7)	16 (39.0)
Treatment-related	7 (16.7)	3 (7.3)
Serious AE	11 (26.2)	12 (29.3)
Treatment-related	3 (7.1)	0
Discontinued study drug due to an AE	3 (7.1)	5 (12.2)
Treatment-related	3 (7.1)	2 (4.9)
Discontinued study drug due to a serious AE	1 (2.4)	3 (7.3)
Treatment-related	1 (2.4)	0
Death	1 (2.4)	1 (2.4)
Treatment-related	0	0

The relatedness of an AE to study drug was determined by the investigator. “Discontinued study drug due to an AE” means that one or more study drugs was discontinued due to an AE

*Abbreviation*: *AE* Adverse event

^a^Non-serious AEs up to 30 days of last dose and serious AEs up to 90 days of last dose are included

^b^AEs (any grade) reported in ≥ 20% of patients in either treatment arm are presented

### Pharmacodynamic activity of epacadostat

**Fig. 3:**
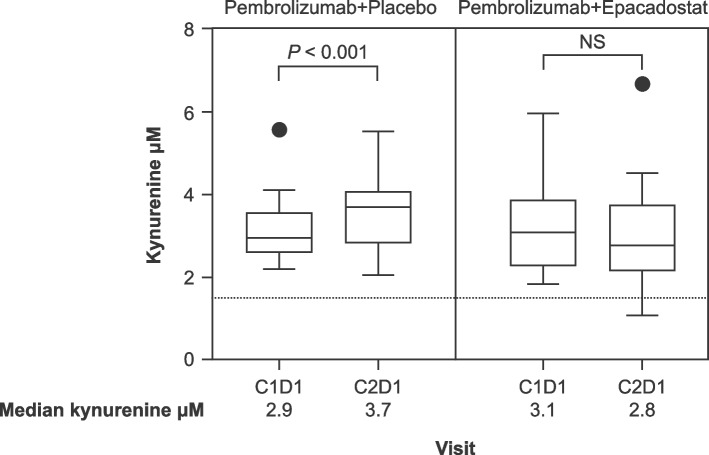
Pharmacodynamic effect of epacadostat 100 mg twice-daily dosing as shown by change from baseline in circulating kynurenine levels. The number of samples assessed was 32 in the pembrolizumab-plus-placebo group and 29 in the pembrolizumab-plus-epacadostat group. Statistical analyses were conducted using paired t-tests within each treatment arm. The dotted line indicates the median kynurenine level in healthy subjects (1.5 μM) [29]. C, Cycle; D, Day; NS, Not significant

Circulating kynurenine levels at baseline (C1D1) and after one cycle of treatment (C2D1) are shown in Fig. 3. Median kynurenine levels increased in the pembrolizumab monotherapy group between cycle 1 and cycle 2 (2.9 µM vs. 3.7 µM) and were similar in the pembrolizumab-plus-epacadostat group between cycle 1 and cycle 2 (3.1 µM vs. 2.8 µM). In both treatment arms, median kynurenine levels at each time point were above the median level in healthy subjects (1.5 µM) [29].

## Discussion

In the ECHO-303/KEYNOTE-698 study of patients with unresectable locally advanced or recurrent/progressive metastatic UC for whom first-line platinum-based chemotherapy failed, the combination of epacadostat plus pembrolizumab yielded an ORR (unconfirmed) that was numerically higher than that observed with pembrolizumab monotherapy (26.2% vs. 11.9%). The ORR for pembrolizumab monotherapy in the current study (11.9%; 95% CI 4.67–29.50) was numerically lower than that observed in the phase III KEYNOTE-045 trial (21.1%) in a similar population [11]. The reasons for this may have been due to the small study size with 95% CIs that overlap with the 95% CI from KEYNOTE-045 and/or the short follow-up in the current study (the ORR may have increased with additional time on study; median duration of pembrolizumab treatment was 65 days in the current study vs 3.5 months in KEYNOTE-045). The combination of epacadostat plus pembrolizumab was generally tolerable in second-line treatment of patients with advanced UC, with a safety profile similar to pembrolizumab alone. No new safety concerns were identified for either treatment, although the proportion of patients with a treatment-related grade ≥ 3 AE was numerically higher with the combination regimen (16.7% vs. 7.3%). The proportion of patients who discontinued study drug due to a treatment-related AE was similar in both treatment arms.

Although no formal statistical testing was undertaken, the findings of the current study show a similar, albeit numerically higher efficacy of epacadostat plus pembrolizumab compared with pembrolizumab monotherapy in second-line treatment of metastatic UC. However, the role of epacadostat for the treatment of advanced UC and other cancers remains unclear based on the negative results of two other phase III studies. In ECHO-307/KEYNOTE-672, epacadostat plus pembrolizumab yielded an ORR (unconfirmed) similar to that observed with pembrolizumab monotherapy in cisplatin-ineligible patients with previously untreated locally advanced or metastatic UC (31.8% and 24.5%, respectively) [34]. In ECHO-301/KEYNOTE-252, no statistically significant differences between epacadostat plus pembrolizumab and placebo plus pembrolizumab were found on the dual primary endpoints in patients with advanced melanoma (median PFS, 4.7 vs. 4.9 months, one-sided *P* = 0.52; median OS, not reached in either arm) [33]. The results of ECHO-301/KEYNOTE-252 led to the premature termination of enrollment to ECHO-307/KEYNOTE-672, as well as to the present study (ECHO-303/KEYNOTE-698) and other ongoing studies exploring combination treatment with epacadostat 100 mg BID and pembrolizumab.

We found that circulating kynurenine levels increased in response to pembrolizumab monotherapy. We hypothesize this observation could be due, at least in part, through elevated IDO1 expression via IFN-y [21]. The addition of epacadostat 100 mg BID to pembrolizumab abrogated this increase but did not normalize the circulating kynurenine values to levels seen in healthy individuals, as was previously reported for epacadostat monotherapy at doses of 100 mg BID or higher [29]. Consistent with this observation, a retrospective pooled analysis showed that epacadostat doses < 600 mg BID were insufficient to maintain suppression of kynurenine production when combined with PD-1 inhibition [35]. Overall, these findings suggest that pembrolizumab-induced kynurenine production was not sufficiently suppressed with the 100 mg BID epacadostat dose evaluated in this trial. Evaluation of higher doses of epacadostat in combination with PD-1 pathway inhibition are warranted, provided that kynurenine can be adequately controlled.

The main limitations of the current study are its small sample size (*N* = 84), short duration of follow-up (median, 62 days in both arms), lack of independent central review of imaging to confirm ORR (introducing the potential for bias), and lack of formal statistical analysis. These factors limit the conclusions that can be drawn from numerical differences between treatment arms and the generalizability of the results from the study.

Additionally, although patient and disease characteristics were generally balanced between treatment arms, a lower proportion of patients in the epacadostat-plus-pembrolizumab arm presented with visceral disease (73.8% vs. 83.3%) or liver metastases (11.9% vs. 21.4%) at baseline relative to the control arm.

## Conclusions

Despite the small sample size and short duration of follow-up of ECHO-303/KEYNOTE-698, epacadostat 100 mg BID plus pembrolizumab demonstrated anti-tumor activity and was generally tolerable in patients with unresectable locally advanced or recurrent/progressive metastatic UC for whom first-line platinum-based chemotherapy failed. More data are needed to evaluate the potential benefit of combined inhibition of IDO1 and PD-L1 in this patient population, and further study of this treatment approach (including testing higher doses of epacadostat) may be warranted.

### Supplementary Information

**Additional file 1:** **Supplementary Table 1.** Investigator-assessed best overall response per RECIST v1.1 based on data acquired only at the week 9 visit (intent-to-treat population).

**Additional file 2: Supplementary Figure 1.** Best target lesion change from baseline based on investigator assessment per RECIST v1.1 and data acquired only at the week 9 visit (intent-to-treat population). **a:** Epacadostat plus pembrolizumab. **b:** Placebo plus pembrolizumab.

## Data Availability

Access to individual patient-level data is not available for this study.

## Abbreviations

AE: Adverse event
BID: Twice daily
C: Cycle
CI: Confidence interval
CPS: Combined positive score
D: Day
IDO1: Indoleamine 2,3-dioxygenase 1
ORR: Objective response rate
OS: Overall survival
PD-1: Programmed cell death protein 1
PD-L1: Programmed death-ligand 1
PFS: Progression-free survival
RECIST v1.1: Response Evaluation Criteria in Solid Tumors version 1.1
UC: Urothelial carcinoma
