# Supplementary material for: Epacadostat plus pembrolizumab versus placebo plus pembrolizumab for advanced urothelial carcinoma: results from the randomized phase III ECHO-303/KEYNOTE-698 study
Source: BMC Cancer. 2024 Jul 25;23(Suppl 1):1256. doi: 10.1186/s12885-023-11213-6 (PMC11270759; doi:10.1186/s12885-023-11213-6)
Supplement: Supplementary file 1 — Additional file 1: Supplementary Table 1. Investigator-assessed best overall response per RECIST v1.1 based on data acquired only at the week 9 visit (intent-to-treat population). [file 12885_2023_11213_MOESM1_ESM.docx]

**Supplementary Table 1.** Investigator-assessed best overall response per RECIST v1.1 based on data acquired only at the week 9 visit (intent-to-treat population)

| ***n* (%)** | **Epacadostat + pembrolizumab**  **(*n* = 42)** | **Placebo + pembrolizumab**  **(*n* = 42)** |
| --- | --- | --- |
| ORR^a^ [95% CI^b^] | 9 (21.4)  [12.88–44.36] | 4 (9.5)  [3.20–26.74] |
| Complete response | 0 | 1 (2.4) |
| Partial response | 9 (21.4) | 3 (7.1) |
| Stable disease | 12 (28.6) | 9 (21.4) |
| Progressive disease | 13 (31.0) | 22 (52.4) |
| No assessment^c^ | 8 (19.0) | 7 (16.7) |

^a^ Includes patients with an unconfirmed complete or partial response

^b^ Per the Clopper-Pearson exact method

^c^ Includes patients with a baseline, but no post-baseline assessment, including those who discontinued or died before the first post-baseline scan

CI, Confidence interval; ORR, Objective response rate; RECIST v1.1, Response Evaluation Criteria in Solid Tumors version 1.1
