# Supplementary figures and images for: Epacadostat plus pembrolizumab versus placebo plus pembrolizumab for advanced urothelial carcinoma: results from the randomized phase III ECHO-303/KEYNOTE-698 study
Source: BMC Cancer. 2024 Jul 25;23(Suppl 1):1256. doi: 10.1186/s12885-023-11213-6 (PMC11270759; doi:10.1186/s12885-023-11213-6)

**A**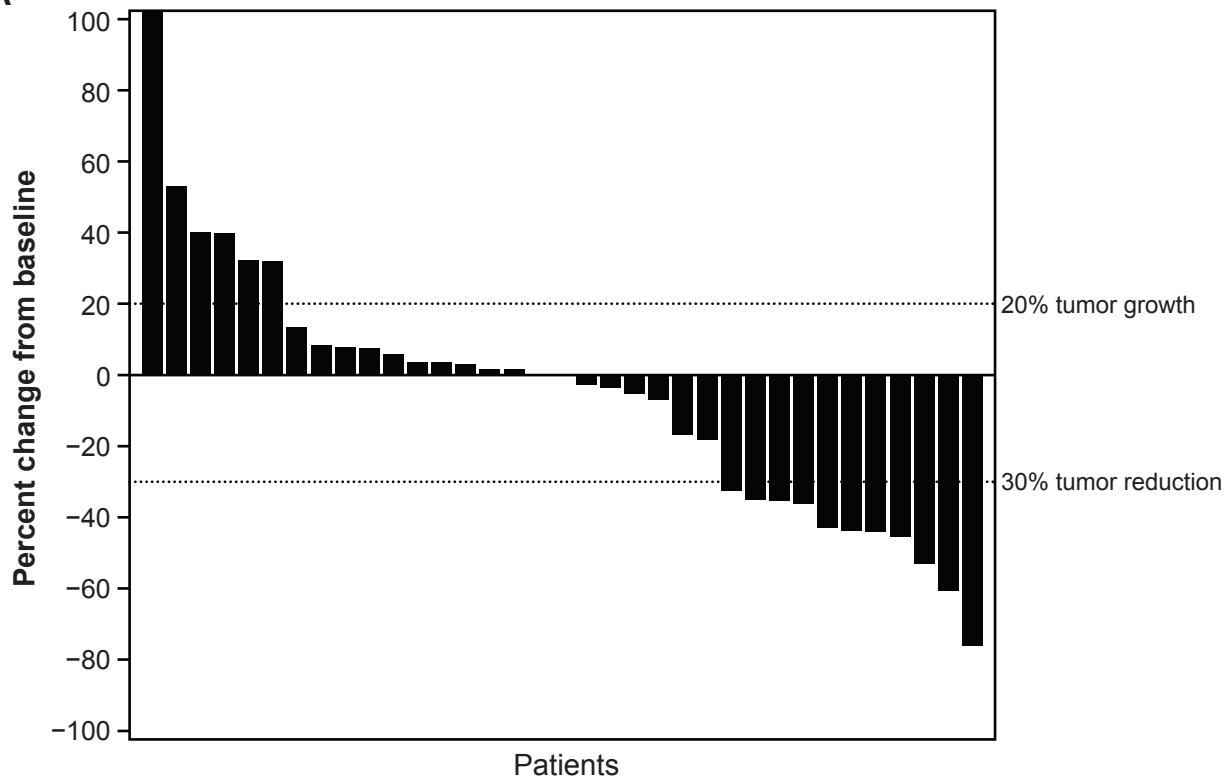**B**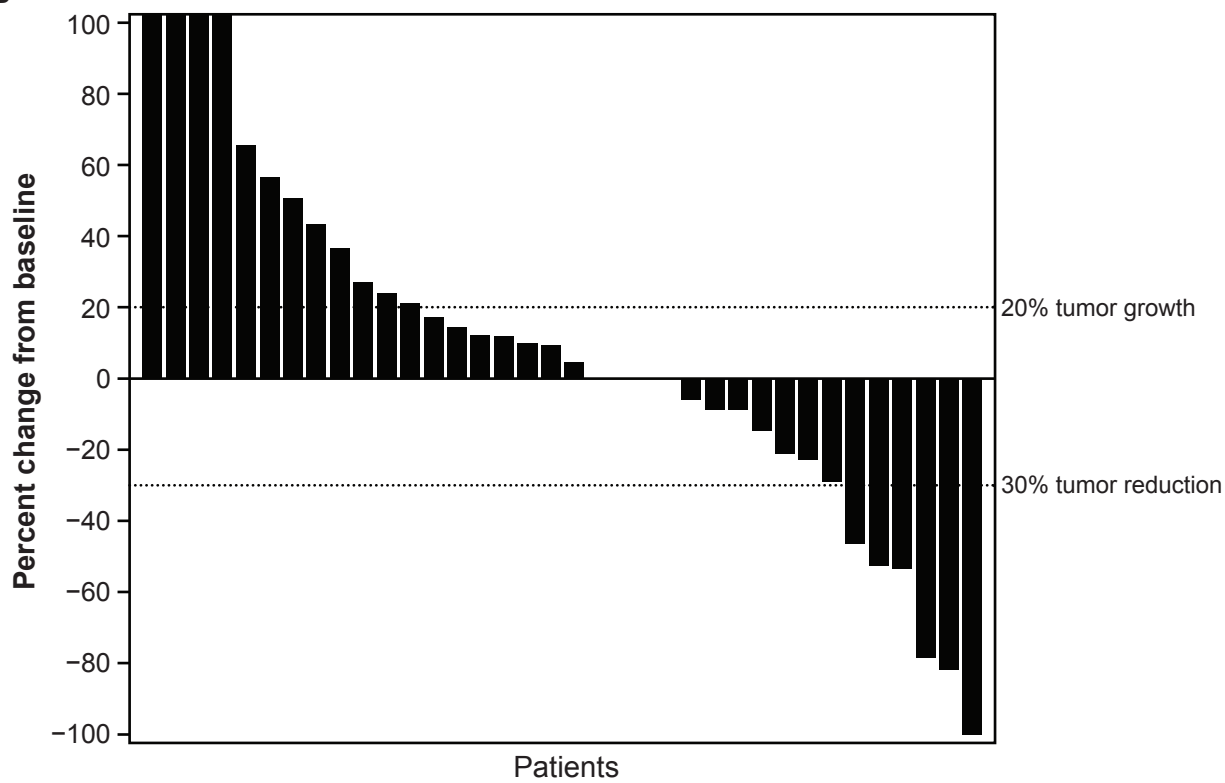

Supplement: Supplementary file 2 — Additional file 2: Supplementary Figure 1. Best target lesion change from baseline based on investigator assessment per RECIST v1.1 and data acquired only at the week 9 visit (intent-to-treat population). a: Epacadostat plus pembrolizumab. b: Placebo plus pembrolizumab. [file 12885_2023_11213_MOESM2_ESM.pdf]
